# Oleanolic Acid (OA) Targeting UNC5B Inhibits Proliferation and EMT of Ovarian Cancer Cell and Increases Chemotherapy Sensitivity of Niraparib

**DOI:** 10.1155/2022/5887671

**Published:** 2022-01-06

**Authors:** Zhen Zeng, Jing Yu, Zhongqing Jiang, Ningwei Zhao

**Affiliations:** ^1^Department of Physiology and Pathophysiology, School of Basic Medical Sciences, Fujian Medical University, Fuzhou 350122, China; ^2^Department of Gynaecology, Affiliated Fuzhou First Hospital of Fujian Medical University, Fujian Medical University, Fuzhou 350009, China; ^3^China Exposomics Institute, Shanghai 201203, China

## Abstract

**Objective:**

To investigate the effect of OA on proliferation, migration, and epithelial-mesenchymal transition (EMT) of ovarian cancer cells by inhibiting UNC5B and to study its mechanism.

**Methods:**

TCGA database was used to analyze the expression of UNC5B in ovarian cancer and its relationship with prognosis. The expression of UNC5B in ovarian cancer cells was detected by qPCR assay. qRT-PCR was used to detect the changes of EMT markers after different treatments. CCK-8 assay was used to detect cell proliferation, transwell assay was used to evaluate cell migration, and clonogenesis assay was used to evaluate the effect of UNC5B on ovarian cancer cell proliferation. Meanwhile, the synergistic effect of OA on niraparib was evaluated.

**Results:**

UNC5B was highly expressed in ovarian cancer, and its expression was negatively correlated with the prognosis of ovarian cancer patients. UNC5B was highly expressed in ovarian cancer cells SKOV3 and OVCA420 compared with normal ovarian epithelial cells. In addition, silencing UNC5B inhibits the proliferation, invasion, clonogenesis, and EMT processes of ovarian cancer cells. OA inhibits proliferation, invasion, and clonogenesis of ovarian cancer cells by inhibiting UNC5B and increases the antitumor activity of niraparib.

**Conclusion:**

UNC5B acts as an oncogenic gene in ovarian cancer. OA inhibits ovarian cancer cell proliferation, migration, and EMT by targeting UNC5B and increases the antitumor effect of niraparib. UNC5B is expected to be a new potential therapeutic target for ovarian cancer. OA may be used as an antitumor drug and deserves further study.

## 1. Introduction

Ovarian cancer is one of the common malignant tumors of female reproductive organs [1–3]. Due to the lack of early diagnosis and the susceptibility to early metastasis, the mortality rate of intermediate and advanced ovarian cancer has remained high [4, 5]. Epithelial to mesenchymal transition (EMT) is an important mechanism of tumor metastasis and invasion [6–8]. EMT refers to the transition of polar epithelial cells from tightly joined epithelial to mesenchymal forms under certain conditions. When EMT occurs, cells lose polarity, intercellular adhesion is weakened, and motor function is enhanced [9, 10]. The expression of epithelial cell markers such as E-cadherin was decreased, while the expression of stromal cell markers such as vimentin was increased. Tumor cells with mesenchymal transformation are less sensitive to existing chemicals. Meanwhile, the ability to invade and migrate to surrounding tissues and distant organs is enhanced [11–13]. This cellular biological behavior is closely related to the occurrence of clinical drug resistance and metastasis. Several studies have demonstrated that inhibition of the EMT process inhibits both metastasis and invasion of ovarian cancer cells [14–16].

UNC5B is a cell membrane receptor belonging to the netrin-1 family of receptors [17]. UNC5B plays an important guiding role in nerve and vascular development [18]. UNC5B expression is associated with vascular formation in hepatocellular carcinoma. Downregulation of UNC5B may promote angiogenesis in hepatocellular carcinoma. The binding of UNC5B to netrin-1 has been shown to inhibit apoptosis in breast cancer [19], lung cancer [20], neuroblastoma [21], and renal ischemic injury [22]. However, the role of UNC5B in ovarian cancer needs to be further studied.

Oleanolic acid (OA) is a kind of natural plant extract and the most widely pentacyclic triterpenoids in nature [23]. In recent years, studies have shown that OA can inhibit tumor growth in liver cancer [24], leukemia [25], lung cancer [26], and other malignant tumors. Previous studies have shown that OA has many benign effects, such as antitumor, liver protection, hypoglycemic, lipid-lowering, immune regulation, anti-inflammatory, antioxidant, and so on [27]. At present, OA preparation is mainly used to treat various types of hepatitis and prevent liver damage caused by antituberculosis drugs. OA has the pharmacological characteristics of high efficiency and low toxicity [28]. However, the mechanism and influencing factors of OA against ovarian cancer are not very clear. Currently, drug therapy is the main clinical treatment for ovarian cancer, but long-term use of first-line drugs for ovarian cancer is likely to cause a variety of adverse reactions and lead to drug resistance of tumor cells [29]. The effect of drug treatment and prognosis of patients are seriously affected. Therefore, it is a great significance to find safe and effective drugs for the prevention and treatment of ovarian cancer. Niraparib is a novel PARP inhibitor and the first targeted drug for recurrent ovarian epithelial carcinoma [30]. Niraparib has a good therapeutic effect on patients with wild-type and mutant types of breast cancer susceptibility gene (BRCA), as well as in combination with radiotherapy and chemotherapy [30, 31].

In this study, the difference of UNC5B expression in ovarian and normal ovarian tissues was analyzed by in vivo and in vitro. At the same time, the effect of UNC5B on ovarian cancer cell proliferation and invasion was verified. In addition, OA inhibited the proliferation and migration of ovarian cancer cell lines by inhibiting the expression of UNC5B. Meanwhile, the mechanism of the synergistic effect of OA on niraparib antitumor activity was studied.

## 2. Methods

### 2.1. Ovarian Cancer Clinical Sample Collection

From January 2020 to January 2021, tumor tissues and adjacent normal tissues of 20 patients with ovarian cancer admitted to our hospital were collected. All specimens were confirmed by 2 pathologists. The patients aged from 39 to 74 years, with an average age of (49.34 ± 4.56) years. Inclusion criteria: (1) histopathologically confirmed EOC. (2) Specimens collected without radiation or chemotherapy. (3) The patients themselves or their authorized family members signed informed consent and voluntarily participated in the study. Exclusion criteria: (1) complicated with serious dysfunction of vital organs such as the heart, lung, liver, and kidney. (2) Complicated with other types of malignant tumors. This study has been approved by the hospital ethics committee.

### 2.2. Cell Culture

Human ovarian cancer cells (Shanghai Cell Bank, CAS) were routinely cultured in a complete medium. The medium contained the RPMI 1640 medium (GIBCO, USA) containing 10% newborn calf serum, 100 U/mL penicillin, and 100 mg/mL streptomycin. Culture in 37°C in a 5% CO_2_ incubator (model 3111, Forma Company of the United States). When the cell density reached 70–80%, the subculture was carried out. Logarithmic growth phase tumor cells were used as experimental cells.

### 2.3. Cell Transfection

1 × 10^6^/mL cells were inoculated in 6-well culture plates. Transfection was carried out when the cell growth reached 50% fusion degree. According to the instructions of Lipofectamine^TM^ 2000 transfection kit, sh-NC and sh-UNC5B were transfected into ovarian cancer cells, respectively. The transfection efficiency was detected after 48 h culture.

### 2.4. Bioinformatics Analysis

The expression of UNC5B in ovarian cancer and normal tissues was analyzed by GEPIA website (http://gepia.cancer-pku.cn/) [32]. The expression and prognostic significance of UNC5B in human ovarian cancer were analyzed by the Human Protein Atlas database [33]. At the same time, through The Human Protein Atlas database (https://www.proteinatlas.org/), obtain UNC5B in normal ovarian tissue and immunohistochemical staining results in ovarian cancer tissue.

### 2.5. qRT-PCR

The TRIzol method extracts RNA from tissues and cells. TransScript® II One-Step gDNA Removal and cDNA Synthesis SuperMix Kit and TransStart® Tip Green qPCR SuperMix Kit were used for RNA reverse transcription and real-time fluorescent quantitative polymerase chain reaction. GAPDH, F: 5′-GGCTGAGAACGGGAAGCTTGTCAT-3′, R: 5′-CAGCCTTCTCCATGGTGGTGGTGAAGA-3′. E-cadherin, F: 5′-AGGAATCCAAAGCCTCAGGT-3′, R: 5′-ACCCACCTCTAAGGCCATCT-3′. Vimentin, F: 5′-GGAAGAGAACTTTGCCGTTG-3′, R: 5′-TCCAGCAGCTTCCTGTAGGT-3′. UNC5B upstream primer 5′-GAGCCGAAACCGCTAATG-3′, downstream primer 5′-CTGCCACTCCAAATGTGATAGA-3′. Use GAPDH as an internal reference. Reverse transcription reaction system: RNA 2 *μ*g, primer 1 *μ*L, reaction mix 2 *μ*L, enzyme mix 2 *μ*L, DNA remover 2 *μ*L, and add enzyme-free water to 20 *μ*L. Reversal conditions: 25°C, 10 min; 50°C, 15 min; 85°C, 5 s. qPCR reaction system: template 4 *μ*L, primer 0.8 *μ*L, reaction solution 10 *μ*L, and double distilled water 5.2 *μ*L. Reaction conditions: 94°C preheating, 100 s; 94°C, 5 s; 60°C, 30 s, 40 cycles; 4°C, unlimited time. Three repeated experiments were performed for each target gene, and the experimental results were processed by the 2^−∆∆Ct^ method.

### 2.6. Transwell Assay

A transwell cell lined with Matrigel (1 : 3) was used to verify cell invasion. Add 200 *μ*L fetal bovine serum medium containing 1 × 10^5^ cells/mL into the upper chamber. Add 600 *μ*L medium containing 20% fetal bovine serum to the lower chamber. Culture at 37°C for 48 h. Remove cells from the upper compartment that have not penetrated the membrane with a cotton swab. The submembrane cells were fixed with 3.7% formaldehyde for 10 min. 0.5% crystal violet was dyed for 5 min. The transmembrane cells in 5 fields were counted under a 200 times microscope, photographed, and the average number of cells was calculated.

### 2.7. CCK-8 Assay

Cells were collected 24 h after transfection in each group. The cells were inoculated into 96-well culture plates at a density of 1 × 10^4^/mL. Culture at 37°C and 5% CO_2_ for 72 h. Each hole is equipped with 3 repeat holes. CCK-8 reagent was added 4 h before the detection time point and incubated for 3 h under dark conditions. After incubation at 37°C for 4 h, the *D* value of each well at 450 nm was measured with a microplate reader. The experiment was repeated three times.

### 2.8. Clone Formation Assay

The effect of UNC5B on cell clonogenesis was investigated by plate cloning assay. This assay was divided into two groups: shRNA-NC group (negative control) and shRNA-UNC5B group. Transacted SKOV3 cells (3 × 10^3^ cells/well) were inoculated into 6-well plates. The growth of cells was observed daily under a light microscope. Change the fresh culture medium 3 days once. 12 days later, the culture was stopped after the formation of clones. Remove the culture medium and wash the cells with PBS for 3 times. Fix with 4% paraformaldehyde solution for 15 min and dye with crystal violet for 24 h. Count the number of clones formed.

### 2.9. Subcutaneous Graft Tumor Model

SPF nude mice, 4–6 weeks old, weight 18–2 g. A total of 12 were obtained, 6 in each group. SKOV3 cells of sh-NC and sh-UNC5B in logarithmic growth phase were taken. After digestion and centrifugation, the samples were suspended in the serum-free RPMI 1640 medium. The cell density was adjusted to 2 × 10^7^/mL. In a sterile environment, cell suspension was prepared by taking the cell lines with good growth and the ratio of 1 : 3 normal saline for injection. 0.1 mL was subcutaneously inoculated in the right axilla of each mouse to prepare an animal model. Four weeks later, carbon dioxide euthanized the nude mice. The tumor tissue was completely exfoliated, weighed, and the tumor inhibition rate was calculated. Animal experiments are approved by the ethics committee of the hospital.

### 2.10. Statistical Analysis

All experiments were repeated 3 times and averaged. SPSS 19.0 statistical software was used for statistical analysis and processing of experimental data. GraphPad Prism 7 (LaJolla, CA, USA) was used. Measurement data were expressed as mean ± standard deviation. The unpaired *t*-test was used for comparison between the two groups. Comparison between multiple groups was analyzed using ANOVA. *P* < 0.05 was considered statistically significant.

## 3. Results

### 3.1. UNC5B is Highly Expressed in Ovarian Cancer and Is Negatively Correlated with Prognosis of Ovarian Cancer Patients

**Figure 1:**
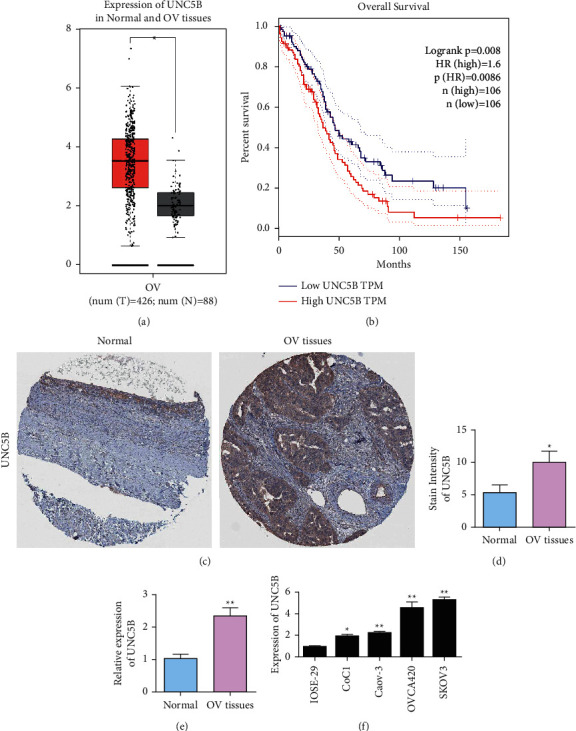
The upregulation of UNC5B in ovarian cancer is associated with poor prognosis of ovarian cancer. (a) Big data analysis of UNC5B expression in normal and ovarian cancer patients. (b) Survival analysis of ovarian cancer patients with different UNC5B expression levels. (c) UNC5B immunohistochemical test results in normal ovarian tissue and ovarian cancer tissue. The experimental results were obtained from the Human Protein Atlas website (http://www.proteinatlas.org/). (d) The statistical results of immunohistochemical detection of UNC5B in normal ovarian tissue and ovarian cancer tissue. (e) RT-qPCR analysis of UNC5B expression in tissues. (f) UNC5B expression level in normal ovarian epithelial cells and ovarian cancer cells. ^*∗*^*P* < 0.05; ^*∗∗*^*P* < 0.01.

The expression of UNC5B in ovarian cancer was analyzed by the TCGA database. Retrieval results showed that UNC5B expression was significantly elevated in ovarian cancer (426 serous ovarian cancer) compared with normal ovarian tissue (*n* = 88) (Figure 1(a)). The relationship between the expression of UNC5B and overall survival (OS) time in ovarian cancer patients was analyzed by the GEPIA database (http://gepia.cancer-pku.cn/). Compared with the group with low UNC5B expression (106 cases), the OS stage of patients with high UNC5B expression (106 cases) was significantly shortened (*P*=0.008) (Figure 1(b)). Immunohistochemistry results showed that the positive rate of UNC5B in ovarian cancer tissues was significantly higher than that in normal adjacent tissues, with a statistically significant difference (Figures 1(c) and 1(d)). Immunohistochemical results were obtained from the Human Protein Atlas website. The expression of UNC5B in ovarian cancer and adjacent normal tissues was analyzed by qRT-PCR. The results showed that UNC5B was overexpressed in ovarian cancer tissues (Figure 1(e)). qRT-PCR results showed that UNC5B protein was highly expressed in CoC1, CAOV-3, OVCA420, and SKOV3 cells compared with IOSE-29 cells (Figure 1(f)). These results suggest that high UNC5B expression is associated with poor prognosis in ovarian cancer patients, and patients with high UNC5B expression have a poor prognosis. These results suggest that UNC5B may be associated with the occurrence and progression of ovarian cancer.

### 3.2. shRNA Silencing UNC5B Expression Inhibits Proliferation, Invasion, and Clonogenesis of Ovarian Cancer Cells

**Figure 2:**
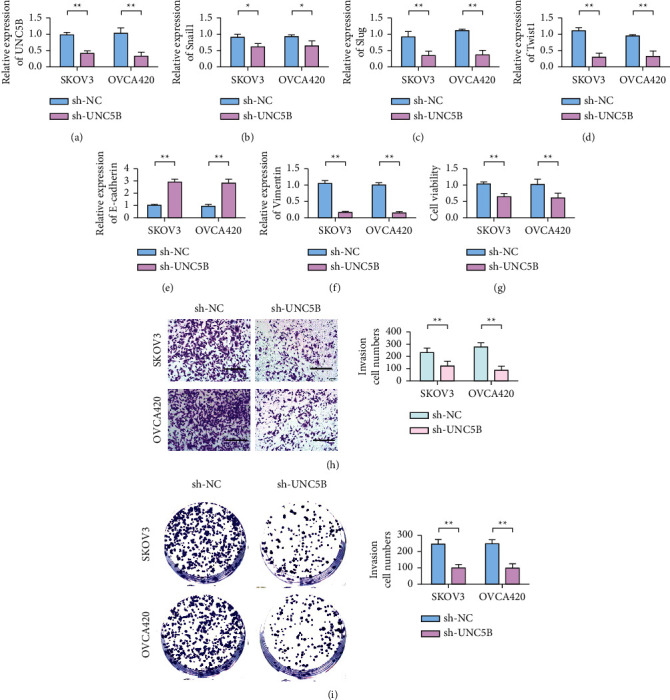
Knockdown of UNC5B inhibits EMT of ovarian cancer cells. (a) Verification of the efficiency of knocking down UNC5B in SKOV3 and OVCA420 cells. (b) Detection of Snail1 expression in SKOV3 and OVCA420 cells. (c) Slug expression detection in SKOV3 and OVCA420 cells. (d) Detection of Twist1 expression in SKOV3 and OVCA420 cells. (e). Detection of E-cadherin expression in SKOV3 and OVCA420 cells. (f) Detection of vimentin expression in SKOV3 and OVCA420 cells. (g) Knockdown of UNC5B inhibits the proliferation of ovarian cancer cells SKOV3 and OVCA420. (h) Knockdown of UNC5B inhibits the invasion of ovarian cancer cells SKOV3 and OVCA420. (i) Knockdown of UNC5B inhibits the clonogenic ability of ovarian cancer cells SKOV3 and OVCA420. ^*∗*^*P* < 0.05; ^*∗∗*^*P* < 0.01.

qRT-PCR results showed that compared with the sh-NC group, the expression level of UNC5B in SKOV3 and OVCA420 cells transferred to sh-UNC5B was significantly downregulated (Figure 2(a)). In this study, SKOV3 and OVCA420 cells transfected with shRNA-UNC5B to stably silence UNC5B expression were constructed. Subsequently, the expression levels of EMT-related transcription factors and markers E-cadherin and vimentin were detected by qRT-PCR before and after different treatments. The results showed that the expression levels of transcription factors for Snail1, Slug, and Twist1 decreased after UNC5B knockdown compared with the sh-NC group (Figures 2(b)–2(d)). Knockdown of UNC5B upregulated E-cadherin expression while inhibiting vimentin expression (Figures 2(e) and 2(f)). CCK-8 assay results (Figure 2(g)) showed that, compared with the sh-NC group, the proliferation ability of SKOV3 and OVCA420 cells was decreased after transferring sh-UNC5B. Transwell assay results (Figure 2(h)) showed that the invasion number of SKOV3 and OVCA420 cells decreased after knockdown of UNC5B. Results of plate clone formation experiment (Figure 2(i)) showed that the number of clone formation of SKOV3 and OVCA420 cells decreased after knockdown of UNC5B.

### 3.3. Silencing UNC5B Inhibits Proliferation and EMT of Ovarian Cancer Cells

**Figure 3:**
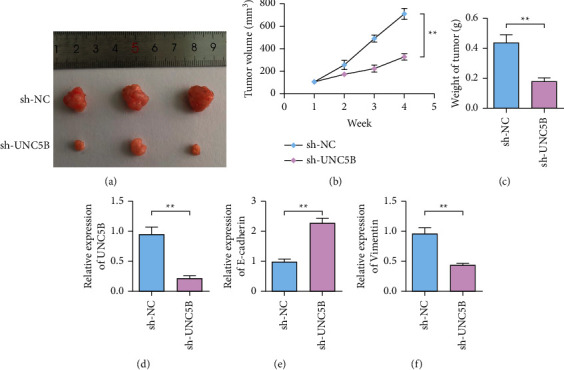
Knockdown of UNC5B gene inhibits the growth of ovarian cancer tumors. (a) Representative pictures of tumors in the control group and UNC5B knockdown group. *N* = 6. (b) Detection of the tumor volume of SKOV3 cells transfected with sh-UNC5B. (c) Detection of the tumor weight of SKOV3 cells transfected with sh-UNC5B and sh-NC. (d) Detection of the expression of UNC5B in the tumor tissue after sh-UNC5B transfected SKOV3 cells are tumor-bearing. (e) Detection of the expression of E-cadherin in the tumor tissue after sh-UNC5B transfected SKOV3 cells are tumor-bearing. (f) Detection the expression of vimentin in tumor tissues after sh-UNC5B transfected SKOV3 cells are tumor-bearing. ^*∗∗*^*P* < 0.01.

The biological effect of UNC5B on ovarian cancer was further verified by tumor-bearing assay in nude mice. In animal studies, knockdown of UNC5B inhibited tumor proliferation in a tumor-bearing model of ovarian cancer (Figures 3(a) and 3(b)). Meanwhile, tumor weight test results showed that tumor weight decreased after silencing UNC5B compared with the sh-NC group (Figure 3(c)). The expression of UNC5B was decreased in ovarian cancer tissues after silencing UNC5B expression compared with the shRNA-NC group (Figure 3(d)). Compared with the shRNA-NC group, the expression of E-cadherin in ovarian cancer tissues after silencing UNC5B expression was upregulated (Figure 3(e)), while expression of vimentin was downregulated (Figure 3(f)).

### 3.4. OA Inhibits the Proliferation, Invasion, and Clonogenesis of Ovarian Cancer Cells by Inhibiting the Expression of UNC5B

**Figure 4:**
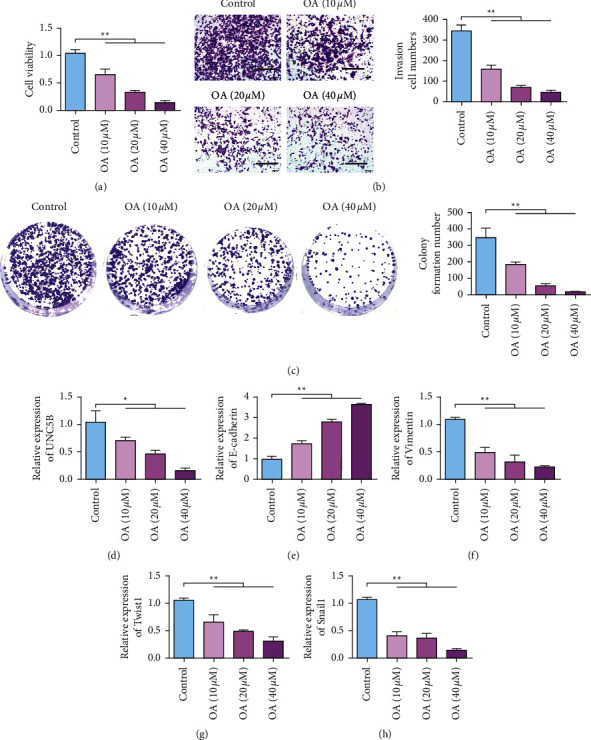
OA inhibits UNC5B and the malignant progression of ovarian cancer cells. (a) After different treatments, SKOV3 cell proliferation detected. (b) Transwell detection of SKOV3 cells after different treatments. (c) After different treatments, the clone formation ability of SKOV3 cells was tested. (d) qRT-PCR detecting the changes in UNC5B expression in SKOV3 cells after different treatments. (e) qRT-PCR detecting the change of E-cadherin expression in SKOV3 cells after different treatments. (f) qRT-PCR detecting the changes of vimentin expression in SKOV3 cells after different treatments. (g) Detection of Twist1 expression in SKOV3 cells after different treatments by qRT-PCR. (h) qRT-PCR detection of Snail1 expression in SKOV3 cells after different treatments. ^*∗*^*P* < 0.05; ^*∗∗*^*P* < 0.01.

The effect of different concentrations of OA on SKOV3 cell growth results showed that SKOV3 cell proliferation ability was significantly reduced after treatment with different concentrations of OA (Figure 4(a)). SKOV3 cells were treated with different concentrations of OA, and their invasion ability and clonogenesis ability were significantly decreased (Figures 4(b) and 4(c)) in a dose-dependent manner. The expression of UNC5B in SKOV3 cells decreased with the increase of OA concentration, in a dose-dependent manner (Figure 4(d)). After 24 h treatment with different doses of OA, the expression of epithelial cell marker E-cadherin was upregulated in SKOV3 cells (Figure 4(e)), while the expression of stromal cell marker vimentin was downregulated (Figure 4(f)). In addition, the expression levels of transcription factors Twist1 and Snail1 also decreased with increasing OA dose (Figures 4(g) and 4(h)). These results indicated that OA could inhibit EMT in ovarian cancer cells.

### 3.5. Silencing UNC5B Expression Can Enhance the Antitumor Activity of Niraparib

**Figure 5:**
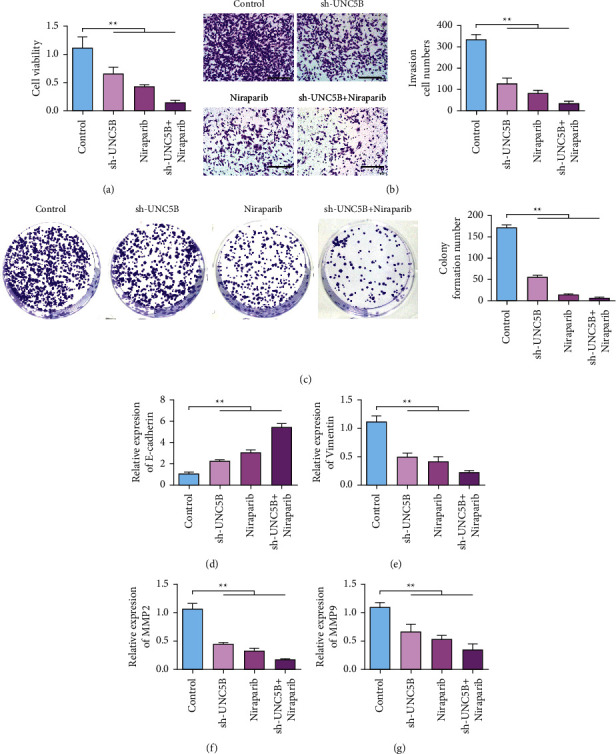
Knockdown of UNC5B can increase the sensitivity of ovarian cancer chemotherapy. (a) After different treatments, SKOV3 cell proliferation detected. (b) Transwell detection of SKOV3 cells after different treatments. (c) After different treatments, the clone formation ability of SKOV3 cells was tested. (d) qRT-PCR detecting the changes of E-cadherin expression in SKOV3 cells after different treatments. (e) qRT-PCR detecting the change of vimentin expression in SKOV3 cells after different treatments. (f) qRT-PCR detecting the changes in MMP2 expression in SKOV3 cells after different treatments. (g) qRT-PCR detecting the change of MMP9 expression in SKOV3 cells after different treatments. ^*∗∗*^*P* < 0.01.

CCK-8 results (Figure 5(a)) showed that the activity of SKOV3 cells in the sh-UNC5B and niraparib groups was decreased. Cells transfected with sh-UNC5B combined with niraparib showed the lowest cell viability. Results of transwell cell migration assay (Figure 5(b)) showed that the invasion number of SKOV3 cells in the sh-UNC5B and niraparib groups was reduced. The number of invasive cells was the lowest in sh-UNC5B combined with the niraparib group. Results of clone formation assay (Figure 5(c)) showed that the number of clones in SKOV3 cells was reduced in sh-UNC5B and niraparib treatment groups. The number of cell clones in sh-UNC5B combined with the niraparib group was the least. EMT marker assay results showed that knockdown of UNC5B or niraparib upregulated the expression of E-cadherin. The expression of E-cadherin was the highest in sh-UNC5B combined with the niraparib group (Figure 5(d)). Knockdown of UNC5B or niraparib inhibits vimentin expression. Vimentin expression was lowest in sh-UNC5B combined with the niraparib group (Figure 5(e)). MMP2 and MMP9 expressions were inhibited by knockdown of UNC5B or niraparib. Meanwhile, MMP2 and MMP9 expression levels were lowest in sh-UNC5B combined with the niraparib group (Figures 5(f) and 5(g)).

### 3.6. The Combination of OA Can Enhance the Antitumor Activity of Niraparib

**Figure 6:**
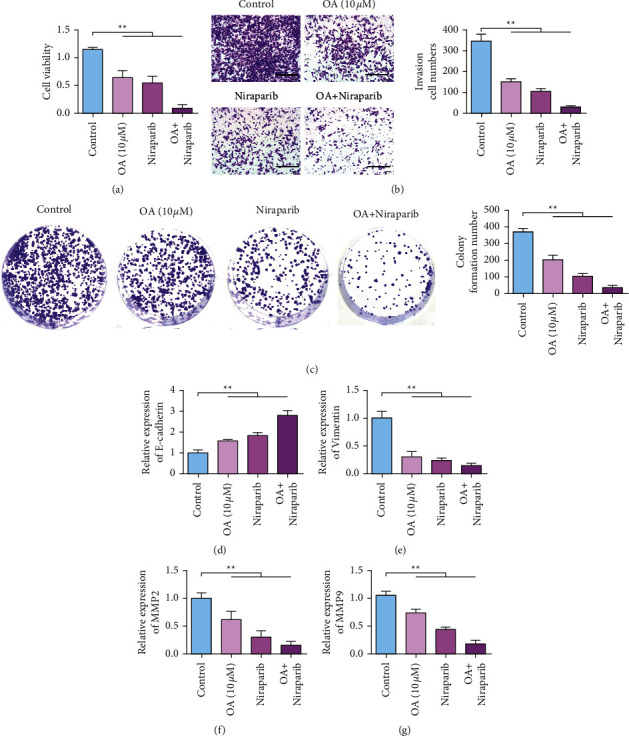
OA increases ovarian cancer chemotherapy sensitivity by inhibiting UNC5B. (a) After different treatments, SKOV3 cell proliferation detected. (b) Transwell detection of SKOV3 cells after different treatments. (c) After different treatments, the clone formation ability of SKOV3 cells was tested. (d) qRT-PCR detecting the changes of E-cadherin expression in SKOV3 cells after different treatments. (e) qRT-PCR detecting the change of vimentin expression in SKOV3 cells after different treatments. (f) qRT-PCR detecting the changes in MMP2 expression in SKOV3 cells after different treatments. (g) qRT-PCR detecting the change of MMP9 expression in SKOV3 cells after different treatments. ^*∗∗*^*P* < 0.01.

CCK-8 results (Figure 6(a)) showed that the activity of SKOV3 cells in the OA treatment group and niraparib treatment group decreased. The cell viability of the combined OA and niraparib group was the lowest. Results of transwell cell migration experiment (Figure 6(b)) showed that the invasion number of SKOV3 cells in the OA treatment group and niraparib treatment group decreased. The number of invasive cells was the least in the combination of OA and niraparib group. Results of clone formation assay (Figure 6(c)) showed that the number of clones in SKOV3 cells treated with OA and niraparib decreased. The number of cell clones in the combination of the OA and niraparib group was the least. EMT marker assay results showed that knockdown of UNC5B or niraparib upregulated the expression of E-cadherin. The expression of E-cadherin was the highest in the simultaneous combination of the OA and niraparib group (Figure 6(d)). Knockdown of UNC5B or niraparib inhibits vimentin expression. The expression level of vimentin in the combination of the OA and niraparib group was the lowest (Figure 6(e)). MMP2 and MMP9 expressions were inhibited by knockdown UNC5B or niraparib. Meanwhile, MMP2 and MMP9 expression levels were the lowest in the group of OA and niraparib (Figures 6(f) and 6(g)).

## 4. Discussion

A large number of studies have proved that the invasion and migration of tumors are related to the occurrence of EMT [34]. The invasion and migration of ovarian cancer cells were significantly enhanced after EMT, and the process of EMT was correlated with acquired drug resistance [6]. Therefore, to elucidate the mechanism of invasion and migration of ovarian cancer has become an urgent problem to overcome ovarian cancer at present, and it is also crucial to improve the prognosis of patients [35].

Ovarian cancer cells tend to infiltrate into surrounding tissues and invade local lymph nodes and the circulatory system in early stage, leading to local implantation metastasis and distant organ metastasis [36]. Blocking EMT of ovarian cancer cells has become a new approach in the treatment of ovarian cancer [37]. A variety of TCM molecular and chemotherapeutic drugs have demonstrated tumor inhibition [38, 39]. Emodin, for example, inhibits ovarian cancer SKOV3 cell invasion by inhibiting EMT by regulating the GSK-3*β*/*β*-catenin/ZEB1 signaling pathway [40]. Sunitinib inhibits the invasion and metastasis of ovarian cancer SKOV3 cells by downregulating the TGF-*β*-mediated EMT process [41, 42].

UNC5 family of homologous proteins, as receptors for secreted protein netrin-1, and its ligand are highly expressed in the nervous system. UNC5 is involved in the development and differentiation of neurons and the directional growth of axons and is closely related to the guiding role of axons [43]. UNC5 homologous proteins are also widely expressed outside of the nervous system. UNC5 plays a role in angiogenesis [18, 44], cell movement, apoptosis [45], and tumorigenesis [46]. UNC5B has been found to be expressed in colorectal cancer, and its expression level is associated with prognosis and recurrence [47]. In this study, qRT-PCR was used to detect the expression of UNC5B in ovarian cancer tissues and adjacent normal tissues. The results showed that the expression level of UNC5B in tumor tissues was significantly higher than that in adjacent normal tissues. These results suggest that UNC5B may be associated with the development of ovarian cancer. Snail1 directly binds to E-cadherin promoter e-boxes and inhibits the expression of E-cadherin. E-cadherin is the most important marker of epithelial cells and is closely related to the formation of EMT [48–50]. The results of this study confirmed that Snail1 expression, epithelial marker E-cadherin expression, and interstitial marker vimentin expression were significantly downregulated after interference with UNC5B expression. Therefore, UNC5B may affect EMT through Snail1 expression, thereby promoting ovarian cancer migration.

In studies on liver cancer, OA can not only block cells in G1 phase but also block a large number of liver cancer cells in Hep3B in G2/M phase. Recent studies suggest that OA can inhibit the proliferation of tumor cells by destroying the active site of the enzyme. The mechanism may be that OA can destroy DNA synthesis and cause tumor cells to be largely blocked during S phase [51]. Apoptosis is an active process to remove the cells that have been damaged and have the tendency to become malignant. OA can induce apoptosis of tumor cells. OA can regulate the expression ratio of Bcl-2 family-related apoptotic proteins to induce apoptosis of HCC cells [52]. OA can also inhibit the motility and adhesion of human lung cancer cells. OA can also inhibit the transmembrane invasion ability of human lung cancer cells. The mechanism is not only to block one process of tumor cell invasion but also to inhibit multiple processes (adhesion, motility, and degradation), probably through inhibition of cell adhesion molecules [53]. In this study, UNC5B may be the target of OA. OA can inhibit the expression of UNC5B and inhibit the proliferation and metastasis of ovarian cancer. Polyadenosine diphosphate ribose polymerase (PARP) is involved in DNA replication and transcription and works with breast cancer susceptibility genes (BRCA) to repair DNA mutated in cells. PARP inhibitors inhibit DNA damage repair in tumor cells, causing DNA damage to accumulate and ultimately kill tumor cells. In this study, OA in combination with niraparib significantly increased the inhibitory effect of niraparib on ovarian cancer.

In this study, the pathway and method of UNC5B affecting ovarian cancer cell migration were investigated. Although the function and mechanism of UNC5B in ovarian cancer have been preliminarily studied in this study, there are still some deficiencies. In this study, the results of transgenic mice and drug combination in vivo were lacking. In addition, animal experiments will be more convincing to show the function of oleanolic acid. The next step will be to further refine the above experiments.

## 5. Conclusion

This study shows that UNC5B is highly expressed in ovarian cancer tissues compared with normal ovarian tissues. UNC5B expression is correlated with prognosis. Interference with UNC5B expression was found to inhibit EMT in vitro studies. Therefore, UNC5B has a potential value as a marker and therapeutic target for ovarian cancer. Furthermore, OA can increase the antitumor activity of niraparib by inhibiting the expression of UNC5B.

## Data Availability

The analyzed datasets generated during the study are available from the corresponding author upon request.
